# Spontaneous Task Structure Formation Results in a Cost to Incidental Memory of Task Stimuli

**DOI:** 10.3389/fpsyg.2019.02833

**Published:** 2019-12-17

**Authors:** Christina Bejjani, Tobias Egner

**Affiliations:** ^1^Department of Psychology and Neuroscience, Duke University, Durham, NC, United States; ^2^Center for Cognitive Neuroscience, Duke University, Durham, NC, United States

**Keywords:** cognitive control, attention, memory, structure learning, cognitive flexibility

## Abstract

Humans are characterized by their ability to leverage rules for classifying and linking stimuli to context-appropriate actions. Previous studies have shown that when humans learn stimulus-response associations for two-dimensional stimuli, they implicitly form and generalize hierarchical rule structures (task-sets). However, the cognitive processes underlying structure formation are poorly understood. Across four experiments, we manipulated how trial-unique images mapped onto responses to bias spontaneous task-set formation and investigated structure learning through the lens of incidental stimulus encoding. Participants performed a learning task designed to either promote task-set formation (by “motor-clustering” possible stimulus-action rules), or to discourage it (by using arbitrary category-response mappings). We adjudicated between two hypotheses: Structure learning may promote attention to task stimuli, thus resulting in better subsequent memory. Alternatively, building task-sets might impose cognitive demands (for instance, on working memory) that divert attention away from stimulus encoding. While the clustering manipulation affected task-set formation, there were also substantial individual differences. Importantly, structure learning incurred a cost: spontaneous task-set formation was associated with diminished stimulus encoding. Thus, spontaneous hierarchical task-set formation appears to involve cognitive demands that divert attention away from encoding of task stimuli during structure learning.

## Introduction

Humans are characterized by a remarkable degree of cognitive flexibility, allowing us to respond to an identical stimulus in a variety of ways, as a function of context. This flexibility derives from our ability to form, apply, and update “task-sets” that define context-specific stimulus-response rules (e.g., Monsell, 2003). The study of task-sets has, to a large extent, focused on how people maintain and switch between explicitly instructed rules, as in classic cued task-switching studies (e.g., Allport et al., 1994; Rogers and Monsell, 1995; reviewed in Kiesel et al., 2010; Vandierendonck et al., 2010). By contrast, how people learn to form task-sets through trial-and-error learning has received much less attention in the task-switching literature (but see Dreisbach et al., 2006, 2007). Moreover, while a parallel learning literature has examined how individuals infer and identify causal structure (e.g., Gershman and Niv, 2010; Wilson and Niv, 2012; Schapiro et al., 2013), not much attention has been paid to the process of task structure building in terms of its immediate cognitive demands and consequences.

Connecting these distinct but related literatures, we here ask the question: How does learning task structure affect the processing of the stimuli that form the input of the learning process? One potent way to answer this question is through the lens of incidental stimulus encoding, since on-task fluctuations in attention ramify in subsequent memory for task stimuli (e.g., deBettencourt et al., 2017). By measuring incidental memory for task stimuli as a function of the structure learning process via a surprise recognition memory test, we can infer where participants focused their attention. This approach has proved successful in elucidating attentional processing during cued task-switching (Richter and Yeung, 2012; Chiu and Egner, 2016). To our knowledge, no previous study has assessed how the process of building and applying a hierarchical task-set impacts the encoding of individual task stimuli. Importantly, an improved understanding of implicit structure formation via incidental encoding adds further insight into interactions between attention and memory in everyday scenarios.

We therefore sought to examine structure learning through incidental task stimulus encoding. We build on recent studies that have shown that when humans learn stimulus-response associations for multi-dimensional stimuli, they implicitly form and generalize abstract, hierarchical task sets (Badre et al., 2010; Collins and Frank, 2013, 2016a,b; Collins et al., 2014; Collins, 2017; Bhandari and Badre, 2018), sometimes even in the absence of inherent structure and performance advantages. Specifically, we here leverage the design of a recent learning task (Collins and Frank, 2016a) that manipulated how stimulus-action rules were mapped to response keys as a means of promoting structure learning. In this task, participants learn to map four two-dimensional stimuli (blue/green triangle/circle) onto four response buttons. To solve this learning problem, participants can memorize all four stimulus-response associations (“flat mapping”) or form a hierarchical mapping, where one higher-level dimension (e.g., color) acts as a context that cues the task-set and the other dimension (e.g., shape) cues the appropriate response rule. We therefore refer to “structure” when participants discriminate the causal relationship between variables in their environment and organize their responses into a hierarchical task-set.

Whether participants build hierarchical task-sets can be inferred from switch costs (cf. Monsell, 2003): participants are slower and more error-prone when their supraordinate dimension (in the above example, color) switches between trials than when their sub-ordinate dimension (i.e., shape) changes, due to additional processing involved in reconfiguring the task-set (Rogers and Monsell, 1995) and/or overcoming proactive interference from the previous set (Allport et al., 1994). Switch costs should be non-existent for participants who adopt a flat mapping, because if they have simply memorized the four stimulus-response associations, there are no supraordinate or sub-ordinate stimulus dimensions. Any transition from one stimulus to another would simply represent a change in the specific S-R mapping retrieved, but would not constitute a task-switch. By contrast, switch costs should be non-zero for participants who adopt a hierarchical task-set, because a change in the supraordinate stimulus dimension would initiate a change in the task rule that is used to determine the correct response (e.g., Collins and Frank, 2013, 2016a,b; Collins et al., 2014).

**FIGURE 1:**
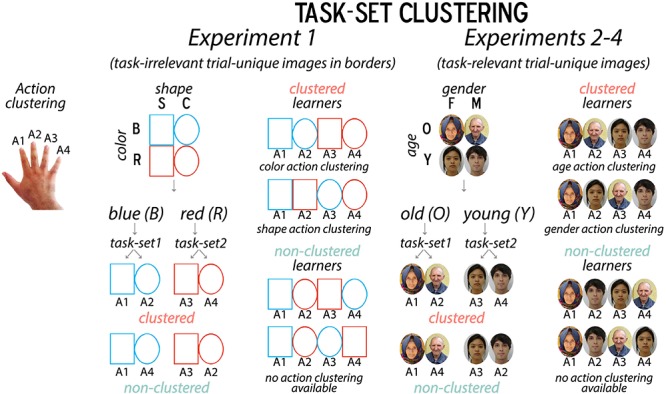
Summary of Task Manipulation. For “non-clustered” learners, response mappings were arbitrarily determined; for “clustered” learners, they were spatially adjacent on the keyboard along a higher-level stimulus dimension. For instance, if all learners applied a hierarchical task-set where color cued the shape task-set, clustered learners would press their right index and middle fingers for a blue square and circle, while non-clustered learners would press their right index and pinky fingers for a blue square and circle. This clustering manipulation was primarily used to promote differences in the strength of structure learning and thus our ability to detect differences in incidental encoding later. Object images were used in Experiment 1, organized by border color (red/blue) and shape (square/circle). Faces were used in Experiments 2–4, organized by apparent gender identity (female/male) and age (young, i.e., less than 30 vs. old, i.e., older than 45).

Given the non-instructed nature of task-set formation processes in this protocol, the experimenter does not have direct control over whether participants adopt a flat mapping or hierarchical task-set strategy in learning the task. However, in order to promote hierarchical set formation in half of the participants and discourage it in the other half, we adopted a biasing technique from Collins and Frank (2016a). Specifically, the proclivity for task-set formation can be biased by manipulating whether stimulus-response mappings are “motor-clustered” along a supraordinate dimension, such that the response mappings for each task-set are either spatially adjacent or not (Figure 1). For example, the color dimension could cue this shape task-set: if participants observe a blue stimulus, they use their right index finger for a triangle and right middle finger for a circle, via a clustered mapping, whereas for a non-clustered mapping, they use their right index finger and right ring or pinky fingers, respectively. This manipulation ultimately results in a greater proclivity for hierarchical structure building (i.e., greater switch costs) for mappings that are motor-biased (i.e., spatially adjacent) than those that are not (Collins and Frank, 2016a), and allows us to naturally bias the likelihood of participants engaging in structure learning.

We here extend this prior work with some key design modifications: we adapt the task to the item (Experiment 1) and category level (Experiments 2–4), using trial-unique object and face stimuli that differ along various stimulus dimensions, so as to assess effects of spontaneous structure learning (as gaged via switch costs) on incidental stimulus encoding in a surprise memory phase (MP). These changes allow us to control for low-level feature priming effects that are inevitable in the original task due to the small stimulus set, and improve ecological validity. Finally, our primary design change allows us to understand structure learning in terms of its immediate cognitive demands and consequences, that is, inferring how implicitly forming task-sets occurs via assessing incidental encoding, which can only be done using trial-unique stimuli. Of particular focus in the context are the early stages of task performance, as this is when the structure learning process would be expected to take place (cf. Badre et al., 2010).

If building spontaneous hierarchical task-sets imposes cognitive demands that divert attention away from stimulus encoding, this would result in worse memory for stimuli encountered, in particular during early structure learning. For instance, structure learning may impose demands on working memory through hypothesis testing of possible rule structures (cf. Gershman and Niv, 2010; Ashby and Maddox, 2011). Alternatively, during learning, participants may selectively attend more to particular stimulus features or dimensions as a means of exploiting environmental redundancy, thus resulting in better subsequent memory for task stimuli (cf. Aly and Turk-Browne, 2017). To adjudicate between these hypotheses, we ran four experiments that individually manipulated the level of stimulus encoding, changes to category-response associations, and instructions about the category-response associations.

## Experiment 1

The goal of Experiment 1 was to replicate the effect of spontaneous task-set formation but with trial-unique stimuli. In this first experiment, we directly adopted the shape/color dimensions employed in previous studies (e.g., Collins and Frank, 2016a) as task-relevant stimulus features, while placing inside the colored shapes trial-unique, task irrelevant object images. This manipulation allowed us to examine how structure learning affects both incidental encoding of these items as well as source memory of the color-shape context in which an item was encountered.

**FIGURE 2:**
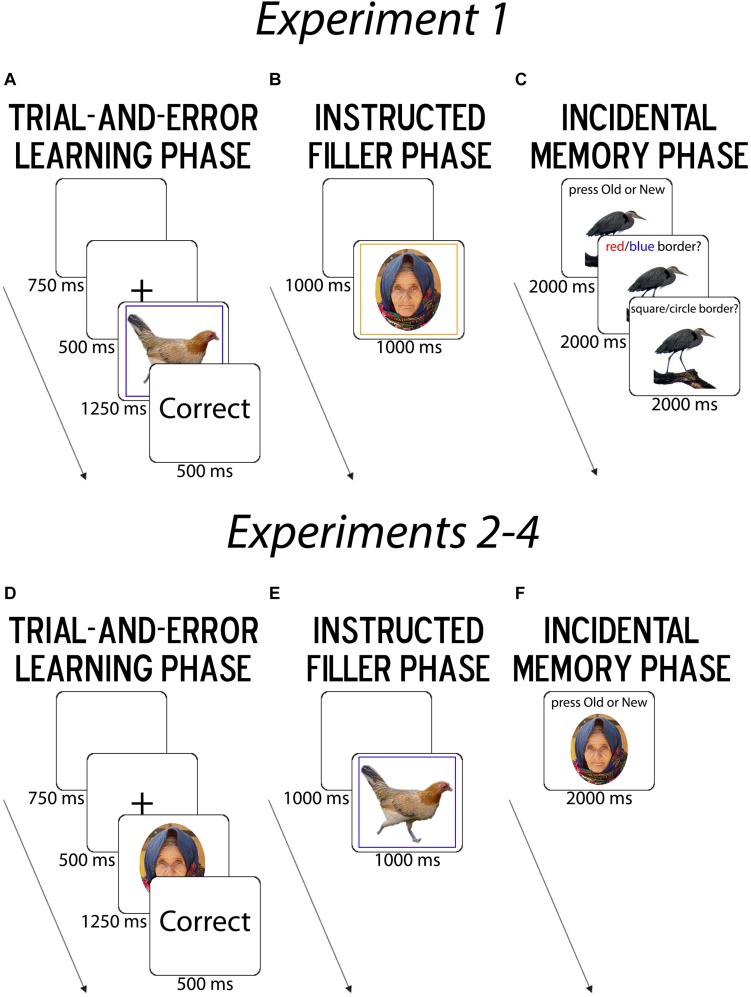
Summary of Task Procedure. **(A,D)** In the trial-and-error learning phase (LP), participants learned the associations between response buttons and image categories via feedback. In Experiment 1, object images were presented within borders that varied by shape (circle/square) and color (red/blue). In Experiments 2–4, face images were presented and varied by age (young/old) and assumed gender identity (male/female). **(B,E)** Next, in the instructed filler phase (FP), participants performed a standard task-switching paradigm in which the color of the border cued the task-relevant judgment, with trial-unique face images [instructed age (young/old) and gender (female/male)] in Experiment 1 and object images [instructed animacy (man-made/natural) and physical size (smaller/larger than a shoebox)] in Experiments 2–4. **(C,F)** Finally, in the incidental memory phase (MP), participants judged whether they had previously seen images in the learning phase. In Experiment 1 alone, because object images were shown within different border colors and shapes, participants were also probed for their source memory of the border color and shape.

The task consisted of a trial-and-error learning phase (LP), an instructed filler task phase, and a surprise MP (Figure 2). In the LP, participants had to learn, via trial-and-error response feedback, which response buttons matched superficial border categories that were characterized by shape (square/circle) and color (blue/red) dimensions and surrounded trial-unique object images. For “non-clustered” learners, response mappings were arbitrary; for “clustered” learners, they were clustered together on the keyboard (v, b, n, m) by stimulus features along a higher-level dimension (e.g., shape or color) (Figure 1). For instance, if participants saw a red square and used color as their higher-level dimension, the corresponding hierarchical task-set that clustered learners should learn was “If red, press v for square and b for circle” while for non-clustered learners the flat mapping, was “If red, press v for square and m for circle.” The motor-clustering manipulation of learner group was primarily used to promote differences in the likelihood and/or strength of structure learning, and this experiment remained closest to the original learning task (cf. Collins and Frank, 2016a), but further tested incidental memory of the task-irrelevant object images. In the filler phase (FP), participants performed a standard instructed task-switching paradigm with different classes of trial-unique stimuli. In the surprise MP, participants were presented with new images and images from the LP, and were first asked to identify whether they had seen the images in the LP and then asked about the rule context in which they had seen the images.

If participants are learning the latent task structure through hypothesis testing, and this process imposes cognitive demands (e.g., on working memory) that divert attention away from the task stimuli, we should observe greater incidental encoding for non-clustered (flat) compared to clustered (hierarchical) learners. However, if participants selectively attend more to particular stimulus dimensions during structure learning, we should observe greater incidental encoding for clustered (hierarchical) compared to non-clustered (flat) learners. For both hypotheses, we predict that differences in memory between the two learner groups should be observed primarily for images that are presented early in the task, when structure learning would be most evident. Clustered learners should take less time than non-clustered learners to acquire category-response mapping associations because of the inherent rule structure (cf. Badre et al., 2010), but once these associations are learned, the learners should perform equally well due to similar attentional and cognitive resources.

### Materials and Methods

#### Participants

Our target sample size was determined based on previous experiments that examined structure learning (Collins and Frank, 2013, 2016a,b; Collins et al., 2014), which had final sample sizes between twenty-two and thirty-five participants. In particular, Collins and Frank (2016a) reported a Cohen’s *d*_*z*_ of 0.88 when comparing motor-clustered RT switch costs against zero, suggesting that for a one-sample two-sided *t*-test with a high power of 0.90 and error rate of 0.05, we would only need sixteen participants to find significant switch costs. Note that in this study, we do not compute a one-sample *t*-test against zero, but instead use permutation-based analyses (cf. Maris and Oostenveld, 2007; Groppe et al., 2011). These analyses are typically more robust and sensitive to trial count in addition to sample size. Moreover, because we were more interested in examining structure building through the lens of incidental memory than probing switch costs, we aimed to recruit around thirty viable participants for each of the two learning groups across our experiments, thus doubling the *a priori* estimated sample size.

Eighty-one Amazon Mechanical Turk (MTurk) workers consented to participate for a $3.85 ($0.13/min) fee in accordance with the policies of the Duke University Institutional Review Board. Nine participants were excluded because of poor accuracy on the LP (<65%; see instruction paragraph below) and nine participants were excluded because of incorrect category-response associations on the post-test questionnaire (see post-test section for more details), resulting in a final sample size of sixty-three (mean age = 32.1, SD = 8.7; 31 female, 32 male; clustered *n* = 31, non-clustered *n* = 32). This level of exclusions is consistent with research suggesting that attrition rates among web-based experiments varies between 3 and 37% (cf. Chandler et al., 2014).

Workers were told the approximate length of the study and the number of the tasks that they had to complete. Workers were asked to take no longer than 4 min for any of the breaks that occurred during the study (e.g., between task phases). Finally, they were also informed that they needed to get above 65% accuracy on the LP for compensation, and that if they got above 90% accuracy, they could earn a flat $1 bonus. Nine workers earned the bonus. Workers who participated in one experiment were explicitly preempted from participating in the others. All exclusion criteria remained the same across experiments.

#### Stimuli

We obtained object images from the Cabeza lab database^1^ and Google searches of images with a license for non-commercial reuse with modification. Pilot data suggested that participants did not spontaneously form task-sets when asked to categorize object images by innate properties such as animacy (man-made/natural) and physical size (smaller/larger than a shoebox), and found these judgments quite difficult. We therefore organized our object images according to superficial stimulus features: border color (red/blue) and shape (square/circle). All object images were cropped to 500 × 300 pixels.

#### Experimental Procedure

The main study manipulation involved motor-clustering biases that encouraged the formation of either a flat or hierarchically structured task-set for shape/color border categories (Figure 1; Collins and Frank, 2016a). This clustering manipulation allowed us to investigate how participants form structured task-sets, shown through switch costs incurred when switching between feature dimension rules that were either superficial (Experiment 1) or inherent to (Experiments 2–4) trial-unique images from higher-level stimulus categories.

The task consisted of consecutive Learning, Filler, and MP (Figure 2). On each trial in the LP (Figure 2A), participants saw a fixation cross for 500 ms and an object image for 1250 ms, followed by performance feedback for 500 ms and a blank ITI screen for 750 ms. Trial-unique object images were shown within a border that varied in color (red/blue) and shape (circle/square), which were the dimensions guiding button responses. Participants were instructed to learn the association between response buttons and border categories (red/blue circle/square) via trial-and-error feedback about their responses. Each response button (v, b, n, and m on a QWERTY keyboard, mapped onto right index, right middle, right ring, and right pinky fingers, respectively) was associated with only one shape/color combination.

To create an encoding-retrieval interval and distract participants from further encoding the LP images prior to the surprise memory test, participants then underwent a 4-minute FP (Figure 2B). The filler task consisted of a standard, cued task-switching protocol Results of this task phase were of no interest to our study goals and are therefore not reported.

Crucially, in the MP (Figure 2C), we then tested whether the learner groups differed in their incidental stimulus encoding and source memory. Participants were shown all the Old images from the LP and ∼1/3 New images (44 due to uneven division of 128 Learning trials), each displayed for 2000 ms. They were then asked whether the borders that had surrounded these images were blue/red or a square/circle, with each source memory question also shown for 2000 ms. The response mappings were always shown on-screen (h, j, k, l mapped to Definitely Old/right index, Probably Old/right middle, Definitely New/right ring, Probably New/right pinky fingers, and a and s mapped to Red/Square/left middle and Blue/Circle/left index fingers, respectively).

For all task phases, if participants did not respond before the image disappeared from the screen, a feedback time-out (“respond quicker”) was provided for 1000 ms to encourage quicker responses. In Experiment 1, this occurred on a total of 3.05% of LP trials, 5.15% of MP trials, and 4.24% and 4.06% of source memory color and shape trials.

All images were presented in the center of the screen. All stimulus categories and trial types were shown in random order, with equal frequency in every task phase [LP: 128 total trials across 1 run; FP: 120 trials across 1 run; MP: 172 trials (128 old/44 new) across 2 runs].

To counterbalance the memory judgments and dimensions around which clustering biases were formed, and manipulate clustering biases, we ran eight task versions. Four versions were essentially duplicates, but with clustered instead of non-clustered motor mappings. We did not have *a priori* hypotheses about whether participants would prefer to use border color/shape (Experiment 1) or age/gender (Experiments 2–4) as supraordinate dimensions, so we ran clustered mapping versions for each dimension. We also varied whether the source memory questions about border color were asked before or after those on border shape.

#### Post-test Questionnaire

After the main experiment, participants filled out a questionnaire that assessed their explicit knowledge of the response mapping and image category associations. If participants could not accurately report the border category-response associations (e.g., whether the button “v” was associated with a “red square” border), we assumed that they either did not learn the associations or were not sufficiently motivated to respond correctly, and they were therefore excluded from the study. Participants also responded to a series of debriefing questions inquiring about their awareness of the task structure. However, responses to these questions largely matched the implicit measures of learned task-switch costs and are therefore not reported here. Finally, participants marked how difficult they found the task on a 1–5 Likert scale, anchored by *not difficult* and *very difficult*, and this measure is not reported here for the same reason.

#### Data Analysis

Analyses were carried out on accuracy (proportion correct) and reaction time (RT) for the LP data and Hit and False Alarm rates for the memory data. Memory data only included trials in which the participants registered a response that was not excessively fast (<200 ms) before the feedback time-out. LP RT was analyzed for correct trials that were not excessively fast (<200 ms) or slow (feedback time-out: >1250 ms). The first trial was excluded for RT and accuracy data for switch cost calculations.

#### Learning Phase Switch Costs

In the Learning Phase, we calculated hypothetical switch costs (Monsell, 2003) to test whether participants formed task-sets according to a higher-level dimension (Collins and Frank, 2013, 2016a,b; Collins et al., 2014). For example, participants could adopt the following task-set structure: “If Red, press v for square and b for circle; if Blue, press n for square and m for circle.” Here, participants would be quicker and more accurate to respond to a red square trial following a red circle trial, or a blue square trial following a blue circle trial, or vice versa (“supraordinate-repeat” trials). If, however, they were forced to switch between the supraordinate red and blue feature rules, they should be slower and less accurate when responding (“supraordinate-switch” trials). Both supraordinate-repeat and switch trials were subtracted from each other to obtain a final switch cost. In calculating switch costs, we excluded trials where there was a double feature switch (e.g., red square followed by blue circle), because these could not be unambiguously attributed to either feature rule.

If participants did not form structured task-sets and instead memorized the associations between buttons and image categories (flat mapping), their switch costs should empirically be zero. Because of our assumption about supraordinate repeat and switch trials, the sign (positive or negative values) of these switch costs only indicated which feature dimension was supraordinate. We therefore took the absolute (unsigned) value of all switch costs and then tested whether participants formed implicit task-sets with a standard permutation method that created an empirical null distribution within each learner group. Specifically, we shuffled the labels of each trial type within each participant, assuming that the conditions were empirically meaningless, and then recalculated switch costs for each participant 10,000 times. For group-level analysis, we compared the mean *z*-score switch cost obtained from our sample, within each learner group, against the 10,000 mean *z*-scores obtained from permutations, testing whether the *z*-score across participants was larger than the *z*-scores generated by an empirical null distribution (two-sided test: 2.5%). For individual difference analysis, we compared the normalized switch cost (d-prime, i.e., $\frac{\mu_{S}-\mu_{N}}{\sqrt{\left(\frac{1}{2}\right)\,\left(\sigma_{S}^{2}+\sigma_{N}^{2}\right)}}$) for each participant against the 10,000 switch costs generated from permutations (two-sided test: 2.5%), testing whether an individual showed a switch cost larger than the switch costs generated by an individual-specific empirical null distribution. We calculated *z*-scores using the standard error of the mean for the group-level analysis, treating all participants as having similar variance, and used standard deviation for the individual difference analysis to account for individual variance. Note that the normalized switch cost does not indicate the strength of structure formation.

Finally, we determined the number of participants who used each feature dimension as a supraordinate rule by evaluating their response mappings and the sign of the raw switch costs.

In sum, we ran permutation tests to determine (a) whether the learner groups showed overall significant switch costs and (b) whether individuals, irrespective of group, showed statistically meaningful switch costs, suggesting spontaneous structure formation. We also determined whether the clustering manipulation was effective by investigating the use of each supraordinate rule and the magnitude of switch costs across groups (see Supplementary Text).

#### Memory

In the MP, we collapsed “Definitely Old” and “Probably Old” and “Definitely New” and “Probably New” responses into aggregate “Old” and “New” measures. We then calculated Hit and False Alarm rates and assessed whether incidental encoding was above chance by comparing Hit and False Alarm rates within each learner group with a paired *t*-test. We also used an independent-samples *t*-test to compare Hit Rates for the one border/image category that had the same response mapping across learner groups (e.g., blue square in Figure 1) as a baseline measure of individual differences in memory capacity or motivation between the groups that might otherwise confound our results.

Exploratory pilot analyses, estimating the trial at which the 95% confidence intervals for performance surpassed chance performance (Smith et al., 2004), suggested that participants took around fifteen trials to learn the associations between response mappings and image categories. However, these individual differences in learning varied by experiment, and depended on the size of the training set for the model (e.g., initial 40 trials vs. all trials). Overall, participants were also more accurate later in the LP, suggesting that after the first sixty or so trials, they had learned the stimulus-response rules.

Thus, to ensure adequate power, we created three bins of 30 and a fourth bin of 38 LP trials, in order to assess learning over the duration of the LP. To assess whether any differences in memory occurred due to time-dependent structure learning, we next ran a repeated-measures ANOVA on Hit rates with LP bin (4) as a within-participants factor and learner group (clustered/non-clustered) as a between-participants factor. In particular, we anticipated differences in memory to be most prominent for images that were shown earlier in the LP, because non-clustered learners should learn the associations between response mappings and border/image categories slightly slower than clustered learners. Thus, we expected early differences in learning to manifest via differences in memory between the learner groups. Because LP bin was a critical factor in our analysis, if participants had fewer than 10 trials in any bin due to a lack of response, they were excluded from the ANOVA. We used this exclusion criterion (i.e., <10 trials) for all memory analyses.

To determine whether source memory was above chance, we compared accuracy for old images rated as new and old with a paired *t*-test for each learner group. We then compared accuracy between learner groups using a repeated-measures ANOVA, with trial type (border color/shape) and rating (old/new) as within-participants factors and learner group (clustered/non-clustered) as a between-participant factor.

In sum, we determined whether participants (a) encoded images above chance, (b) differed in baseline levels of motivation or memory capacity, and (c) showed differences in time-dependent learning across groups.

All data were Greenhouse–Geisser corrected where appropriate. Effect sizes were calculated according to published recommendations (Lakens, 2013)^2^. Data and experimental code are online at https://github.com/christinabejjani/ClusteredTSMem.

### Results

#### Learning Phase (LP)

**FIGURE 3:**
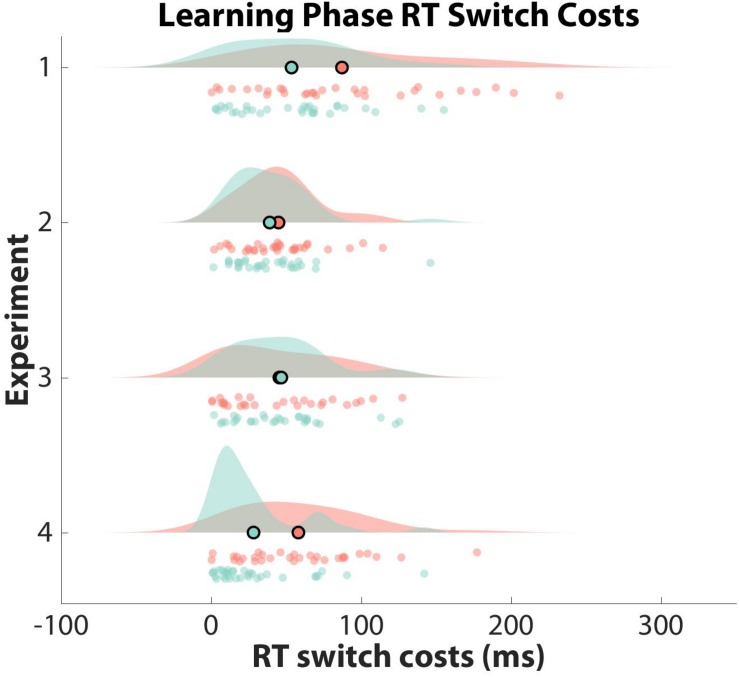
Observed Learning Phase RT Switch Costs. Learning phase reaction time (RT) switch costs are shown as a function of experiment and learner group (clustered: red; non-clustered: green). Switch costs are displayed in milliseconds on the *x*-axis, with the mean of each distribution outlined in black as a scatter plot point. Participant data are displayed as scatter points. Note that only Experiment 2 generated significant switch costs for the clustered learner group.

**FIGURE 4:**
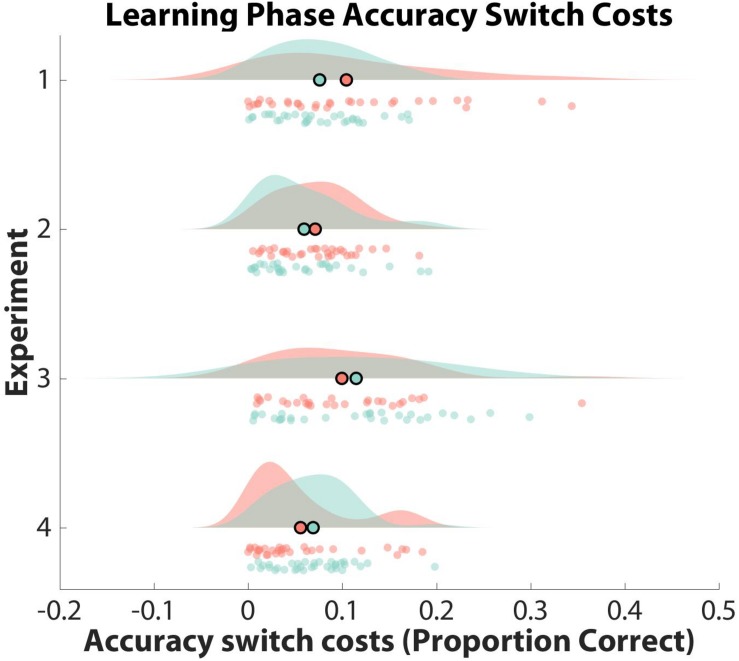
Observed Learning Phase Accuracy Switch Costs. Learning phase accuracy switch costs are shown as a function of experiment and learner group (clustered: red; non-clustered: green). Switch costs are displayed as a proportion of correct responses on the *x*-axis, with the mean of each distribution outlined in black as a scatter plot point. Participant data are displayed as scatter points. Note that only Experiment 2 generated significant switch costs for the clustered learner group.

To test whether participants formed hierarchical task-sets at the group-level, we compared their normalized switch costs against an empirical null distribution. Specifically, we counted the number of mean *z*-score switch costs from the empirical null distribution that were smaller than the mean *z*-score switch cost obtained from our data for both learner groups separately. Mean *z*-score switch costs were not significant (i.e., two-sided test: greater than 97.50%) for clustered learners or non-clustered learners when compared to their respective empirical null distributions (clustered: RT = 68.41%, Accuracy = 16.54%; non-clustered: RT = 59.98%, Accuracy = 92.98%). Thus, neither group showed significant evidence of structure formation. See Figures 3, 4 for RT and accuracy switch cost distributions and Supplementary Table 1 for switch cost means and confidence intervals across all experiments.

To explore individual differences in structure formation, we then compared the normalized switch cost for each participant against their individual-specific null distribution. Thirteen participants in the clustered group and two participants in the non-clustered group had RT or accuracy normalized switch costs that were at least 97.50% larger than their respective permuted switch costs.

Signed mean RT switch costs indicated that 32 participants categorized object images by border color and 31 by border shape (clustered: 18 BC, 13 BS; non-clustered: 14 BC, 18 BS). This suggests two points: (1) participants had about equal likelihood of using either stimulus feature as a supraordinate dimension and (2) most participants in the clustered group followed the mapping suggested by the cluster manipulation.

#### Memory Phase (MP)

Both clustered [Hit vs. FA: *t*(30) = 6.51, *p* < 0.001, Cohen’s *d* = 0.91, CL effect size = 88%] and non-clustered learners [*t*(31) = 4.28, *p* < 0.001, Cohen’s *d* = 0.58, CL effect size = 78%] remembered the task-irrelevant object images above chance.

However, consistent with the lack of group-level structure formation, we observed little evidence for group differences in incidental memory. See Supplementary Table 2 for full behavioral data from the MP across experiments.

**FIGURE 5:**
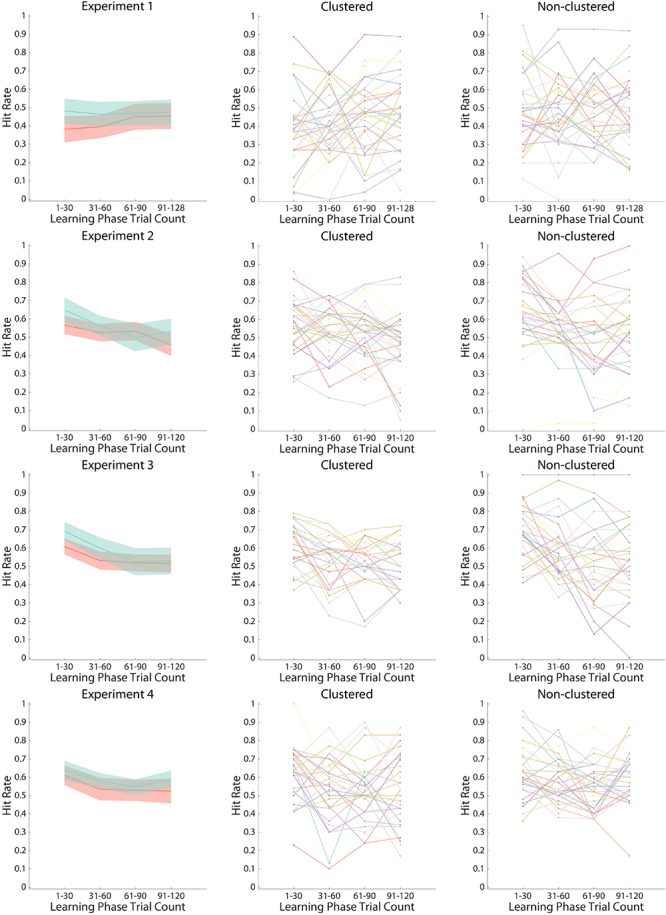
Hit Rates in the Memory Phase. Hit rates are shown as a function of experiment (top row: Experiment 1; bottom row: Experiment 4), learner group (clustered: red line in left panel and all lines in middle panel; non-clustered: green line in left panel and all lines in right panel), and learning phase bins (four bins of thirty images each). The line in the left panel indicates the mean Hit rate across participants within learner groups, with the 95% confidence interval shaded for each group. The center and right panels indicate individual Hit rates, with each individual in a different color.

There were no differences in incidental encoding via time-dependent learning [Figure 5; LP bin: *F*(2.75,167.96) = 1.04, *p* = 0.371, η*_*p*_^2^* = 0.02; LP bin × group: *F*(2.75,167.96) = 1.27, *p* = 0.288, η*_*p*_^2^* = 0.02]. Nor did we observe any differences in overall Hit Rate between learner groups [group: *F*(1,61) = 1.82, *p* = 0.182, η*_*p*_^2^* = 0.03].

Neither clustered learners nor non-clustered learners had above chance source memory accuracy for border color or shape trials (Clustered: Old images rated Old vs. New: *t*s < 0.42; Non-clustered: *t*s < 0.97). There were no significant effects on source memory (*F*s < 1.68).

### Discussion

The results of Experiment 1 did not replicate previous evidence of spontaneous task-set formation (cf. Collins and Frank, 2013, 2016a,b; Collins et al., 2014). Consistent with the lack of learner group differences in spontaneous task-set formation, we also found no significant differences in recognition memory between groups. In fact, overall memory performance was rather poor.

In Experiment 1, the trial-unique images were irrelevant to task-set formation, which may have degraded stimulus processing altogether. Prior studies employed very small sets of task relevant stimulus features (cf. Collins and Frank, 2013, 2016a,b; Collins et al., 2014), which did not allow for tests of recognition memory and may suggest that structure formation is prone to large individual differences or results from feature priming effects across trials. In order to answer our question about how learning task structure impacts incidental stimulus encoding, and whether spontaneous structure formation is constrained by feature priming, we addressed this level of encoding limitation in Experiment 2 by using task-relevant trial-unique images.

## Experiment 2

We ran Experiment 2 to address the question of how structure learning, facilitated by motor clustering biases, affects incidental encoding. Because the object images in Experiment 1 were task-irrelevant, this may have masked differences in how learning affects incidental encoding, and hurt our ability to adjudicate between our hypotheses. Specifically, the effect of structure learning on incidental memory may depend on the level of stimulus encoding. We therefore sought to render the trial-unique images directly task-relevant, which should promote deeper encoding. Including task-relevant trial-unique images should also control for feature priming confounds in previous work, since each trial now shows a completely new and unique stimulus (cf. Collins and Frank, 2013, 2016a,b; Collins et al., 2014).

Faces are social, have inherent value (e.g., Smith et al., 2010), can prime attentional categories (e.g., Cañadas et al., 2013), and may be categorized by a number of features (e.g., age, gender identity, emotion). Here, using face features as task-relevant stimulus categories (face age/gender identity), we tested how implicit task-set learning affects cognitive flexibility and incidental encoding.

### Materials and Methods

#### Participants

Eighty-four MTurk workers consented to participate for a $2.25 ($0.13/min) fee. Two participants were excluded because they were older than 60 (72 and 73); seven participants were excluded for incorrect answers on the post-test assessing their knowledge of the category-response associations; and nine participants were excluded due to poor accuracy (<65%), resulting in a final sample size of sixty-four (mean age = 34.86, SD = 8.62; 27 female, 37 male; clustered *n* = 33, non-clustered *n* = 31). Thirty participants earned the $1 bonus.

#### Stimuli

We obtained face images from several databases (Ebner et al., 2010; Bainbridge et al., 2013; http://fei.edu.br/~cet/facedatabase.html) and Google searches for older men and women under a non-commercial reuse with modification license. Piloting confirmed that the modal response for gender was either female or male and was neutral for emotion (instead of happy, sad, angry, fearful, surprised, or disgusted). The mean and modal response for age indicated that young faces were rated as less than 20 years or 20–30 years old, while old faces were rated as 45–60 years or 60+ years old. We excluded faces that were not neutral, ambiguous in assumed gender identity, 30–45 years old, and celebrity images. We ensured that each stimulus category had exactly 43 white/Caucasian faces and nine faces of other racial categories (Young female: four South Asian, three Hispanic, one East Asian, one Black; Old female: four East Asian, three South Asian, two Middle-Eastern; Young male: five Hispanic, four South Asian; Old male: five Middle-Eastern, two South Asian, two East Asian; race/ethnicity, age, emotion, and gender options mimicked Bainbridge et al. (2013) for consistency). All images were cropped into an oval frame taken from Bainbridge et al. (2013) and were 280 × 350 pixels.

Once we applied these filters to minimize differences in arousal and promote easy categorization of age and gender identity, we had a final stimulus set of 208 face images that differed in age (young, i.e., younger than 30 and old, i.e., older than 45) and gender identity (female/male) but not emotion (all neutral), and was somewhat diverse and controlled for race/ethnicity. This ensured that we had manipulated the stimulus categories that might influence how participants learned structured task-sets.

#### Experimental Procedure

Experiment 2 used face stimuli, but otherwise employed the same task structure as Experiment 1 (Figures 1, 2). Participants were now told that face images varied according to age (young, i.e., less than 30 years old, and old, i.e., more than 45 years old) and gender (female/male) rather than the superficial border categories we used for object images in Experiment 1 (Figure 2D). Here, we used 120 LP trials, keeping a similar trial count to ensure that recognition memory accuracy was high.

For the 160 MP trials (120 old/40 new), Definitely Old, Probably Old, Definitely New, Probably New were mapped onto the a, s, k, and l keys and left middle and index and right index and middle fingers, respectively. Because this experiment required judgments based on inherent rather than artificially created contextual stimulus properties, there were no source memory questions (Figure 2F).

Feedback time-outs occurred on a total of 2.91% of LP trials and 2.92% of MP trials.

There were four task versions, counterbalanced for the biased dimension (age/gender) and learner group (clustered/non-clustered).

### Results

#### Learning Phase (LP)

Mean *z*-score switch costs for clustered learners were significantly larger than *z*-score switch costs derived from the empirical null distribution (RT: 97.83%, Accuracy: 98.11%). For non-clustered learners, these percentages were 75.80% and 29.02%, suggesting that clustered learners, but not non-clustered learners, spontaneously formed structured task-sets.

Examining individual differences in spontaneous task-set formation, we found that two clustered learners and one non-clustered learner showed RT or accuracy normalized switch costs larger than their individual permuted switch costs at least 97.50% of the time.

Reaction time switch costs suggested that 31 participants employed gender and 33 employed age as the supraordinate dimension (clustered: 17 gender, 16 age; non-clustered: 14 gender, 17 age).

#### Memory Phase (MP)

Both clustered [Hit vs. FA: *t*(32) = 6.75, *p* < 0.001, Cohen’s *d* = 1.44, CL effect size = 88%] and non-clustered learners [*t*(30) = 5.78, *p* < 0.001, Cohen’s *d* = 0.96, CL effect size = 85%] remembered the face images above chance. As with Experiment 1, Hit rates for images that had the same response mapping across learner groups did not differ between the learner groups (*t* < 0.04).

In line with time-dependent learning, both learner groups encoded face images presented earlier in the LP better than those presented later [LP bin: *F*(2.26,135.52) = 10.46, *p* < 0.001, η*_*p*_^2^* = 0.15]. However, consistent with our hypothesis that differences in memory would emerge early in the task, when structure learning was most prominent, non-clustered learners had larger Hit rates than clustered learners for earlier images [LP bin × group: *F*(2.26,135.52) = 3.14, *p* = 0.040, η*_*p*_^2^* = 0.05], but not larger Hit rates overall [group: *F*(1,60) = 1.31, *p* = 0.257, η*_*p*_^2^* = 0.02]. A follow-up *t*-test indicated that non-clustered learners showed a marginal trend toward better memory than clustered learners for the first 30 LP trials (*t*(60) = 1.96, *p* = 0.055, Cohen’s *d* = 0.51, CL effect size = 64%; clustered: M = 0.57, 95% CI [0.51, 0.62]; non-clustered: M = 0.65, 95% CI [0.58, 0.72]).

Are any differences in memory related to individual variability in spontaneous task-set formation? We added individual RT and accuracy switch costs as covariates in an exploratory repeated-measures ANOVA, reanalyzing the memory data while accounting for a continuous measure of how individuals’ structure formation affects time-dependent learning. Results were largely in line with the ANOVA at the group-level [LP bin: *F*(2.22,128.64) = 2.26, *p* = 0.103, η*_*p*_^2^* = 0.04; LP bin × group: *F*(2.22,128.64) = 2.84, *p* = 0.056, η*_*p*_^2^* = 0.05; all other *Fs* < 1.22]. Altogether these results suggest that structure learning may slightly degrade early incidental encoding for task-relevant, trial-unique images.

### Discussion

The results of Experiment 2 provide evidence that humans generate hierarchical task-sets for grouping trial-unique stimuli into categories. Although the stimuli were completely new and unique on every trial, controlling for feature priming confounds (cf. Collins and Frank, 2013, 2016a,b; Collins et al., 2014), we found that most clustered, but not non-clustered, learners formed implicit task-sets. To our knowledge, this is the first demonstration of people spontaneously forming hierarchical task sets at the category level, and it qualifies previous conclusions about the nature of spontaneous structure formation (cf. Collins and Frank, 2013, 2016a,b; Collins et al., 2014). When stimuli allow for easy categorization (via motor clustering) and are task-relevant, participants spontaneously build task-sets; otherwise, this tendency toward structure formation may be less common in larger, less superficial stimulus sets.

Consistent with the hypothesis that differences in memory would occur earlier as a function of structure learning, we found (weak) evidence that the tendency toward spontaneous task-set formation resulted in a cost to memory: the early phase of spontaneous hierarchical task-set formation was associated with slightly diminished task stimulus encoding. However, although we found a significant interaction between the time when images were shown in the LP and the clustering manipulation, the evidence regarding differences in memory requires replication, given the small effect size. Therefore, in Experiment 3, we sought to replicate the differences in memory we observed and address more specifically the effect of structure learning on memory.

## Experiment 3

We hypothesize that the differences in memory we observed in Experiment 2 stem primarily from differences in early structure formation. To test this prediction, we next sought to manipulate our task design such that participants would have to learn new category-response associations, but not infer new latent causes or branches for their task-sets. Specifically, this would require remapping the learned associations, but not the latent task structure.

Inspired by the Wisconsin Card Sorting Task (Grant and Berg, 1948), we aimed to investigate whether an implicit switch in S-R rules would alter incidental memory. Specifically, if the response mappings were disrupted, would this lead to differences in later memory between the learners? To this end, in Experiment 3, halfway through the LP, unbeknownst to participants, the supraordinate dimension guiding response mappings was flipped, which forced participants to learn new category-response associations. Because the implicit rule switch occurred halfway through the LP, we anticipated replicating the association between early task-set formation and diminished stimulus encoding.

If differences in memory were caused by differences in attention for particular stimulus-response associations, we should observe differences in later incidental encoding between the groups after the implicit rule switch, especially for the stimuli with response mappings that changed. However, if differences in memory were caused by differences in early structure learning, we should observe no differences in later incidental encoding, even while participants adapt to the implicit rule switch (as that switch did not involve a new task structure).

### Materials and Methods

#### Participants

Eighty-three MTurk workers consented to participate for a $2.25 ($0.13/min) fee. Fourteen participants were excluded because of incorrect post-test identification of the category-response associations, and eleven participants were excluded because of poor accuracy (<65%), resulting in a final sample size of fifty-eight (mean age = 33.24, SD = 8.37; 30 female, 27 male, 1 no reply; clustered *n* = 28 and non-clustered *n* = 30). Nine participants earned the $1 bonus.

#### Experimental Procedure

Experiment 3 used the same stimuli and task structure as Experiment 2, but included an implicit rule switch in the LP. This implicit response mapping switch was foreshadowed in the task instructions. Specifically, the instructions said:

“Please note that the correct response button for each face image category may, in fact, change during the task.”

This is in contrast to:

“The correct response button for each image category will not change during this task.” (Experiment 1)

“Please note that the correct response button for each face image category will not change during the task.” (Experiment 2, Experiment 4)

Halfway into the LP, the response mappings changed. For clustered learners, this was a flip of the motor-clustered supraordinate dimension: response mappings became clustered around the other stimulus feature. Non-clustered learners meanwhile had to relearn a new pair of associations. For both learner groups, this dimensional flip meant that two of the stimulus categories had the same button mapping throughout the task (“positive transfer”), while two changed (“negative transfer”). Although the pre-task instructions hinted at the rule switch, there was no indication while participants performed the task that the mappings had changed beyond the trial-by-trial feedback that they received.

Feedback time-outs occurred on a total of 2.35% of LP trials and 2.27% of MP trials.

First, to assess whether participants were able to adapt to the rule change, despite potentially detrimental transfer costs in accuracy, we compared switch costs for before and after the implicit rule switch, using a repeated-measures ANOVA with time (pre/post rule switch) as the within-subjects factor and learner group (clustered/non-clustered) as the between-subjects factor. Next, to assess whether the rule switch was effective and resulted in transfer costs in accuracy, we ran a repeated-measures ANOVA on the post-switch accuracy data, using transfer (positive/negative) and time bin (61–90, 91–120 trial count) as within-participants factors and learner group (clustered/non-clustered) as the between-participants factor. We predicted that participants would show worse accuracy for negative vs. positive transfer trials, because structure would facilitate performance for positive, but not negative, transfer trials, and that these transfer costs should diminish over time as participants relearned the category-response associations. Analyses otherwise remained the same as in Experiment 2.

### Results

#### Learning Phase (LP)

Mean *z*-score switch costs for clustered learners did not differ significantly from the empirical null distribution (RT: 31.20%, Accuracy: 54.42%). These percentages were similar for non-clustered learners (RT: 74.75%, Accuracy: 63.59%). Results were non-significant when *z*-scored switch costs were calculated using only the sixty trials before the rule switch (clustered RT: 54.43%, Accuracy: 88.65%; non-clustered RT: 55.24%, Accuracy: 88.27%).

When examining individual differences in spontaneous task-set formation, two clustered and five non-clustered learners showed significant RT or accuracy switch costs. When only considering the first sixty trials, this estimate changes to four and three learners, respectively, suggesting that fewer trials for switch cost estimation affected the variance within participants for the overall analysis.

Reaction time switch costs suggest that 31 participants employed gender and 27 employed age as the supraordinate dimension (clustered: 15 gender, 13 age; non-clustered: 16 gender, 14 age).

The implicit rule switch successfully affected performance, as shown by (1) smaller switch costs and (2) worse accuracy when learned associations changed (transfer costs). Absolute mean switch costs were smaller after the rule switch across groups [RT time: *F*(1,56) = 4.32, *p* = 0.042, η*_*p*_^2^* = 0.07; accuracy: *F*(1,56) = 5.02, *p* = 0.029, η*_*p*_^2^* = 0.08; all other effects: *F* < 1.74]. The main effects of transfer [*F*(1,56) = 89.16, *p* < 0.001, η*_*p*_^2^* = 0.61] and LP bin [*F*(1,56) = 58.70, *p* < 0.001, η*_*p*_^2^* = 0.51] on accuracy were qualified by an interaction (*F*(1,56) = 94.77, *p* < 0.001, η*_*p*_^2^* = 0.63). Thus, participants had transfer costs that degraded over time as they learned the new associations (all other effects: *F* < 0.48).

#### Memory Phase (MP)

Both clustered [Hit vs. FA: *t*(27) = 6.55, *p* < 0.001, Cohen’s *d* = 1.66, CL effect size = 89%] and non-clustered learners [*t*(29) = 6.81, *p* < 0.001, Cohen’s *d* = 1.10, CL effect size = 89%] remembered the face images at above chance. As with Experiments 1 and 2, Hit rates for images that had the same response mapping across learner groups (*t* < 0.89) did not differ between the learner groups.

All participants encoded face images presented earlier in the LP better than those presented later [LP bin: *F*(2.41,134.74) = 15.02, *p* < 0.001, η*_*p*_^2^* = 0.21]. This effect did not vary by learner group over the course of the experiment [LP bin × group: *F*(2.41,134.74) = 1.53, *p* = 0.216, η*_*p*_^2^* = 0.03; group: *F*(1,56) = 1.91, *p* = 0.172, η*_*p*_^2^* = 0.03]. When we account for the individual RT and accuracy pre-rule-switch switch costs, the results of the ANOVA remain largely similar [LP bin: *F*(2.42,128.46) = 4.49, *p* = 0.009, η*_*p*_^2^* = 0.08; RT switch cost: *F*(1,53) = 2.81, *p* = 0.100, η*_*p*_^2^* = 0.05; all other *F*s < 1.45]. However, replicating Experiment 2, non-clustered learners showed better memory than clustered learners for the first 30 LP images (*t*(56) = 2.48, *p* = 0.016, Cohen’s *d* = 0.66, CL effect size = 68%; clustered: M = 0.61, 95% CI [0.56, 0.65]; non-clustered: M = 0.69, 95% CI [0.64, 0.74]).

### Discussion

In Experiment 3, unlike in Experiment 2, we did not observe significant group-level switch costs for either learner group, in part because the implicit rule switch successfully disrupted task-set formation for all participants. However, as in Experiment 2, we were able to identify individual participants who showed significant switch costs.

One unanswered question is whether participants kept the same hierarchical task structure or changed their supraordinate rules to adapt to the change in response mappings. Switch costs differed before and after the rule switch, suggesting that most participants kept applying the same task-sets they had originally learned, even though the button mappings had changed. However, the permutation analysis suggested that only some participants formed hierarchical task-sets in the first place. This question could be answered by increasing analytic sensitivity via a larger trial count prior to the implicit rule switch or with an fMRI experiment, probing whether representations of the stimulus categories, perhaps in the prefrontal cortex (Badre et al., 2010), vary as a function of learning and the implicit rule switch (cf. Kriegeskorte and Kievit, 2013).

Finally, we also replicated the association between diminished incidental encoding and early task-set formation. We found that non-clustered learners encoded images presented earlier in the LP better than clustered learners. Because this occurred for Experiments 2 and 3, but not Experiment 1, we infer that this effect is only present when images are encoded relatively deeply – here, in the context of classifying faces by gender or age (or both) – as compared to the completely incidental processing in Experiment 1. Experiment 3 also suggests that this effect is driven by differences in early structure learning: after an implicit rule switch that disrupted category-response associations, but kept the same latent task structure, we did not observe any differences in memory between the learner groups. However, in order to definitively test whether these differences in incidental encoding are truly driven by structure learning rather than differences in attention for particular S-R associations, implicit, trial-by-error task-set learning should be contrasted against the instructed implementation of task-sets. We therefore chose to run an instructed mappings version of Experiment 2 in Experiment 4.

## Experiment 4

To further examine whether differences in trial-and-error task-set learning drove the memory differences between the learner groups, we ran a version of Experiment 2 that pitted implicit task-set learning against the simple implementation of explicitly instructed response mappings (flat or hierarchical). If we observed differences in memory even when the response mappings were explicit and participants no longer needed to learn the association between response buttons and face image categories through trial-and-error, this would indicate that the implementation of structure alone could change incidental encoding. If, however, we observed tempered or no differences in memory between hierarchical vs. flat instructed mappings, this would suggest that interactions between early structure building and associative learning subsequently shape incidental memory.

### Materials and Methods

#### Participants

Eighty-eight MTurk workers consented to participate for a $2.25 ($0.13/min) fee. Seven participants were excluded because of incorrect post-test identification of the category-response associations, and sixteen participants were excluded because of poor accuracy (<65%), resulting in a final sample size of sixty-five (mean age = 33.25, SD = 9.37; 33 female, 32 male; clustered *n* = 31 and non-clustered *n* = 34). Thirty-six participants earned the $1 bonus.

#### Experimental Procedure

Experiment 4 differed from Experiment 2 only in that it explicitly informed participants of the response mappings. For example, instead of reading,

“You will have to figure out, through trial-and-error via feedback about your responses, which button response is correct for each category. You will use the v/V, b/B, n/N, and m/M keys to respond.”

Participants read:

Clustered: “If the face image is YOUNG, press v/V for female and b/B for male. If the face image is OLD, press n/N for female and m/M for male.”

Non-clustered: “If the face image is YOUNG, press v/V for female and m/M for male. If the face image is OLD, press n/N for female and b/B for male.”

Feedback time-outs occurred on a total of 3.38% of LP trials and 2.99% of MP trials.

### Results

#### Learning Phase (LP)

Unlike Experiments 1–3, while mean *z*-score switch costs were not significant for clustered learners when compared to an empirical null distribution (RT: 81.19%; accuracy: 9.09%), accuracy switch costs for non-clustered learners were significant (RT: 0.29%, accuracy: 98.60%). With instructed rather than implicitly learned response mappings, perhaps participants had more difficulty with implementing the task-set rules when they were not intuitively structured, i.e., adjacent on the keyboard.

When examining individual differences, however, ten clustered and one non-clustered learners showed significant RT or accuracy switch costs at least 97.50% of the time, suggesting that differences in structure formation observed at the group-level are subject to much individual variation.

Reaction time switch costs suggested that 34 participants employed gender and 31 employed age as the supraordinate dimension (clustered: 18 gender, 13 age; non-clustered: 16 gender, 18 age).

#### Memory Phase (MP)

Both clustered [Hit vs. FA: *t*(30) = 8.68, *p* < 0.001, Cohen’s *d* = 2.04, CL effect size = 94%] and non-clustered learners [*t*(32) = 7.20, *p* < 0.001, Cohen’s *d* = 1.93, CL effect size = 90%] remembered the face images at above chance level. Unlike Experiments 1–3, Hit rates for images that had the same response mapping across learner groups were slightly higher for clustered learners, suggesting the existence of some individual differences in memory capacity or motivation [*t*(62) = 1.78, *p* = 0.081, Cohen’s *d* = 0.45, CL effect size = 62%].

As with Experiments 2 and 3, both learner groups encoded face images presented earlier in the LP better than those presented later [LP bin: *F*(3,183) = 5.67, *p* < 0.001, η*_*p*_^2^* = 0.09]. Now that the task-sets were instructed, however, this memory difference did not vary by group [LP bin × group: *F* < 0.47; group: *F*(1,61) = 2.04, *p* = 0.158, η*_*p*_^2^* = 0.03]. Indeed, in contrast to Experiments 2 and 3, the learners had similar Hit rates for the first 30 LP images (*t* < 0.72; clustered: M = 0.61, 95% CI [0.56, 0.66]; non-clustered: M = 0.64, 95% CI [0.58, 0.69]).

When we accounted for individual differences in switch costs, results from the ANOVA were largely similar [LP bin: *F*(3,177) = 2.22, *p* = 0.088, η*_*p*_^2^* = 0.04; group: *F*(1,59) = 2.53, *p* = 0.117, η*_*p*_^2^* = 0.04; Accuracy switch cost: *F*(1,59) = 2.12, *p* = 0.151, η*_*p*_^2^* = 0.04; LP bin × group: *F*(3,177) = 1.88, *p* = 0.133, η*_*p*_^2^* = 0.03; RT switch cost *F* < 0.15], although there was a significant interaction between individual accuracy switch cost and LP bin [*F*(3,177) = 2.69, *p* = 0.048, η*_*p*_^2^* = 0.04] and an interaction between individual RT switch cost and bin [*F*(3,177) = 3.57, *p* = 0.015, η*_*p*_^2^* = 0.06]. Individuals with smaller RT and accuracy switch costs had primacy and recency effects in memory, while individuals with larger switch costs showed a strong primacy effect, with average Hit rates largely decreasing in each subsequent bin.

#### Compiling Data Across Experiments

In Experiments 2 and 3, the first sixty trials were identical across the task design, Experiments 2–4 all used face stimuli, and Experiments 2 and 4 differed only in whether participants had to implicitly learn or had instructed task-sets. We therefore performed *post hoc* comparisons across MP data in order to increase our statistical power.

To examine the effect of early task-set formation on Hit Rates, we ran a repeated-measures ANOVA with LP bin (1–30, 31–60) as a within-participants factor and Experiment (2/3) and learner group (clustered/non-clustered) as between-participants factors. Hit rates were higher for the first 30 vs. second 30 images from the LP [bin: *F*(1,116) = 33.47, *p* < 0.001, η*_*p*_^2^* = 0.22], and this did not vary by experiment [bin x group: *F*(1,116) = 1.34, *p* = 0.249, η*_*p*_^2^* = 0.01; experiment: *F*(1,116) = 1.84, *p* = 0.178, η*_*p*_^2^* = 0.02; all other effects, *F* < 0.78]. However, Hit rates for the first 60 LP trials were significantly larger for non-clustered than clustered learners (group: *F*(1,116) = 8.32, *p* = 0.005, η*_*p*_^2^* = 0.07; first 30 images: *t*(118) = 3.10, *p* = 0.003, Cohen’s *d* = 0.57, CL effect size = 66%; clustered M = 0.59, 95% CI [0.55, 0.62]; non-clustered M = 0.67, 95% CI [0.63, 0.71]). Results were similar when we added in the individual RT and accuracy switch costs [group: *F*(1,113) = 6.36, *p* = 0.013, η*_*p*_^2^* = 0.05; bin: *F*(1,113) = 10.44, *p* = 0.002, η*_*p*_^2^* = 0.09; Experiment: *F*(1,113) = 3.14, *p* = 0.079, η*_*p*_^2^* = 0.03; RT switch costs: *F*(1,113) = 2.52, *p* = 0.115, η*_*p*_^2^* = 0.02; all other *F*s < 1.72].

To compare the effects of instructed vs. implicit task-set learning on incidental encoding, we ran a repeated-measures ANOVA on Hit Rates for the first sixty trials from Experiments 2–4, using LP bin (1–30, 31–60) as a within-participants factor and learning type (trial-and-error/instructed) and cluster group (cluster/non-clustered) as between-participants factors. We found, again, that participants encoded the first 30 vs. second 30 LP images better (bin: *F*(1,179) = 35.91, *p* < 0.001, η*_*p*_^2^* = 0.17), and that this did not vary by experiment (all other effects, *F* < 1.13). However, as expected, non-clustered learners encoded the first sixty LP images better than clustered learners (group: *F*(1,179) = 6.98, *p* = 0.009, η*_*p*_^2^* = 0.04; first 30 images: *t*(181) = 2.93, *p* = 0.004, Cohen’s *d* = 0.44, CL effect size = 62%, clustered M = 0.59, 95% CI [0.57, 0.62], non-clustered M = 0.66, 95% CI [0.63, 0.69]), suggesting that structure learning, facilitated by motor-clustering biases, plays a larger role in differences observed for early incidental encoding than any difference between learning implicit vs. instructed task-sets. Results were similar when we added in the individual RT and accuracy switch costs [group: *F*(1,176) = 3.88, *p* = 0.050, η*_*p*_^2^* = 0.02; bin: *F*(1,176) = 13.54, *p* < 0.001, η*_*p*_^2^* = 0.07; RT switch cost: *F*(1,176) = 3.12, *p* = 0.079, η*_*p*_^2^* = 0.02; all other *F*s < 1.29].

### Discussion

In our everyday lives, adaptive behavior is facilitated by our ability to discover and leverage rules for grouping stimuli and linking them to appropriate actions. While previous literature has shown that humans can spontaneously form and generalize abstract rule structures for two-dimensional stimuli, relatively little work has investigated how such structure learning is achieved or how it impacts concurrent stimulus processing and encoding. Here, we leveraged the human tendency to exploit redundancy within complex environments by manipulating response mappings from factorially crossed stimulus categories to bias spontaneous task-set formation.

We first examined switch costs and found that people generate clustered task-sets for grouping trial-unique stimuli into categories when stimulus-response mappings encourage dimensional grouping, but that this tendency toward structure formation may, in fact, be more of an individual difference than a general effect. Specifically, only in Experiment 2 did clustered learners show strong switch costs consistent with spontaneous task-set formation, although there were individuals from both learner groups in all experiments who showed significant switch costs. Because we found significant switch costs within individuals of each group across all experiments, but only found significant switch costs within the clustered learners for Experiment 2, we thus infer that people spontaneously form task-sets, but that the boundary conditions are quite narrow. Specifically, spontaneous task-set formation may be more likely only when there are feature repetitions and a small stimulus set (cf. Collins and Frank, 2013, 2016a,b; Collins et al., 2014).

Second, we found that experimentally clustering the category-response associations increased switch costs, but may not have directly related to biasing spontaneous task-set formation. When the category-response associations were instructed (Experiment 4) rather than implicitly discovered (Experiments 2, 3), and the stimulus dimensions were superficial (Experiment 1) rather than semantic stimulus properties (Experiments 2, 3), clustered learners had larger switch costs than non-clustered learners (see Supplementary Text). Aggregating the data into a larger model across the three experiments that used task-relevant stimulus categories (Experiments 2–4), results suggested that the motor-clustering manipulation increased switch costs more when task-sets were acquired via explicit instructions than through experience. While we mostly do not replicate the main findings of Collins and Frank (2016a), only observing spontaneous task-set formation at the group level when category-response associations were implicitly learned over many trials, we do replicate the “clustering bonus,” or increase in the mean switch cost, associated with the experimental manipulation.

Third, and most importantly, this tendency to build structured task-sets resulted in a cost to memory: the early phase of spontaneous task-set formation was associated with diminished task stimulus encoding. When trial-unique images were task-relevant and deeply encoded (Experiments 2, 3), Hit rates were higher for images presented earlier in the LP for non-clustered vs. clustered learners. Combining data with that of Experiment 4, where the task-sets were explicitly instructed, these memory differences still held. Across four experiments, we found little evidence that individual differences in memory capacity or motivation, measured by Hit rates for the category-response association that was held constant across learner groups, affected our results. In short, because we found an association between worse memory for early incidental encoding of the images and spontaneous task-set formation, we conclude that participants learned the task structure through hypothesis testing, which imposed cognitive demands that diverted attention away from the task stimuli.

An alternative explanation of these findings could be that learning the category-response mappings sapped attention away from the individual images, leading to differences in memory between the learner groups. For instance, although both groups were engaged in learning the category-response associations, clustered learners may have learned the associations quicker and used short cuts once they learned the basic task structure (e.g., “long white hair” means “old female”) rather than process the images holistically. We directly tested this possibility by switching the stimulus dimension around which response mappings were organized (Experiment 3), which disrupted category-response associations without changing the basic causal task structure. We found no differences in memory post-switch between the learner groups and replicated the differences in incidental encoding between the learner groups for images presented earlier in the LP. Although memory was overall poorer post-switch than pre-switch, we also found that across Experiments 2–4, memory was better for both learner groups for task-relevant images presented earlier than later in the LP. These results thus provide evidence that the differences in incidental encoding we observed were caused specifically by differences in early structure learning.

In sum, while learning the category-response associations and building structured task-sets, all participants are likely testing hypotheses about how to best exploit the redundancy in the task environment (cf. Kemp and Tenenbaum, 2009; Gershman and Niv, 2010), including the motor-clustering manipulation. When structure learning is facilitated by motor-clustering biases, it diminishes incidental stimulus encoding. Future research could explicitly test the working memory demands imposed by such implicit task-set formation with the manipulation in Experiment 2, but with occasional working memory probes in the LP instead of the full-fledged three-phase task design used here. If structure learning imposes additional working memory demands, participants should do worse at immediately matching the trial-unique images they were recently categorizing when in the cluster than non-clustered group. The level of specificity within working memory could also be manipulated by probing whether participants still have the particular category template vs. specific image in mind and testing how these working memory probes impact switch costs across groups.

One notable limitation in these experiments, however, is that the spatial distances on the keyboard differ as a function of the clustering manipulation (Figure 1). For the clustered learners, the distances between buttons are on average 1.5 keyboard units (1, 1, 2, 2), while for the non-clustered learners, the distances are on average 2.3 (2, 2, 4, 1). Nonetheless, it is highly unlikely that any differences in recognition memory result as a function of these differences in button distance: participants showed no significant differences in memory for the one category of images that had the same button mapping across learner groups (see Supplementary Table 2). Moreover, not all participants showed evidence of structure formation and the results suggested that memory differences were specific to individuals who showed significant switch costs.

Finally, although we were able to aggregate data across Experiments 2–4, we could not include Experiment 1. This is a notable design limitation: an alternate version of Experiment 1 could have involved face images within superficial borders, manipulating which dimensions were task relevant as a factor across Experiments 1 and 2 (i.e., borders vs. gender/age). That manipulation would have avoided the confound of particular stimulus details and the difficulty we faced in having participants categorize object images.

Similarly, another design limitation is our inability to determine whether spontaneous task-set formation caused, or was associated with, diminished task stimulus encoding. The non-instructed nature of the task-set formation process meant that participants within both learner groups were able to spontaneously learn the latent task structure and that we did not have control over any strategy they used. We therefore do not know what the exact reasons are for a participant adopting hierarchical representations. To facilitate comparison with Collins and Frank (2016a), we also created empirical null distributions for switch costs within cluster groups; for a more generalizable analysis, we could have shuffled the group label to compare switch costs for a random sample of participants.

To understand how this work fits within existing literature, we will now consider three related fields: category learning, control learning, and reinforcement learning (RL).

#### Relationship to Category Learning

Within the category learning literature, two memory systems are described as competing for influence when humans decide on optimal decision rules (Ashby and Maddox, 2005, 2011; for debate on multiple memory systems, see e.g., Poldrack and Foerde, 2008). The verbal system learns explicit, consciously controlled rules through hypothesis testing, while the implicit system uses procedural learning to maximize accuracy on a categorization task by integrating information across stimulus dimensions.

Although the verbal system closely resembles the structured task-set learning outlined in this study, with rules instantiated through reinforcement history until they are no longer useful, we do not view our work through the lens of category learning, for several reasons. First, a key component of the verbal system is that rules are explicit and verbalizable, and our study was not designed to disentangle the effects of conscious awareness. The facial stimulus dimensions were also not separable, and when humans have more difficulty selectively attending to a single stimulus dimension, it is typically assumed that the implicit system wins the competition with the verbal system (Ashby et al., 1998). Finally, in many category-learning tasks, the goal is to acquire abstract knowledge via new categories. Our task explicitly relies on highly learned categories like faces, and increasing levels of expertise with a category (cf. Gauthier et al., 2000) can change stimulus processing, suggesting that our study involves category representations that inform spontaneous task-set formation rather than explicit categorization or category learning *per se*.

However, future research could isolate the similarities and differences between category and task-set learning. For example, it is assumed that there is a bias toward the verbal system, with humans biased to spontaneously form task-sets. Switch costs may reflect the cognitively demanding process of reconfiguring between task-sets (Koch et al., 2018), while category learning also assumes that switching between the categorization systems is subject to interference (Crossley et al., 2018). At what point do these systems differ? Longman et al. (2018) recently found that instructions can bias stimulus-response learning over category-response learning and stimulus-classification learning, suggesting that one point of differentiation may stem from how the learning goal is framed.

#### Relationship to Control-Learning

Task-set learning comprises a subdomain of the control-learning literature. In the most related branch of that literature, participants learn to associate the process of task-switching with predictive contextual cues or task statistics (Dreisbach and Haider, 2006; Leboe et al., 2008; Crump and Logan, 2010; Chiu and Egner, 2017). This type of control-learning differs from most other studies on task-switching, where participants are given explicit cues to recruit control and prepare for an upcoming switch, or a feature of the task stimulus itself cues switching between tasks (Monsell, 2003). However, both research domains have focused on how task-set learning or preparation reflects increased cognitive flexibility (Koch et al., 2018), ignoring how structure learning can bias control-learning (cf. Bejjani et al., 2018).

Recently, Bhandari and Badre (2018) have illustrated how learning abstract task knowledge can facilitate control processing: they found that participants can learn and transfer control policies that support flexible behavior based on the task structure. Such policy abstraction (cf. Collins and Frank, 2013, 2016a,b; Collins et al., 2014) is consistent with how our participants implicitly built task-sets, even when there was no inherent benefit to doing so. This increased flexibility can, unfortunately, result in costs to memory. Both children and adults who perform a learning task in which pairs of objects are associated with particular response rules suffer an accuracy cost when the response rule shifts for the first time for a particular object pair, but readily adapt on subsequent shifts (Darby et al., 2018). Such conflict between long-term encoding of pair-response contingencies and the response rule currently in working memory suggests that there may be a cost to memory incurred with the cognitive flexibility provided by structure building. One question that arises from these data is whether this cost to memory is observed only for the type of structure building assessed in our study or generalizes to scenarios when selective attention more readily biases control processing (e.g., Addleman et al., 2018).

#### Relationship to Reinforcement Learning

The control-learning, category learning, and structure learning literatures are often described in terms of RL. A basic RL problem involves a set of environmental states, a set of actions taken at these states, a transition function that maps how actions will cause the transition to another state, and a reward function that indicates the amount of reward available at each state (Sutton and Barto, 1998). The typical assumption is that agents learn a set of actions, or a *policy*, that maximizes overall reward. However, with larger task domains, the number of actions and states that the agent must track also increases, taxing the system such that basic RL models do not scale well with increasing task complexity (Botvinick et al., 2009). Simple RL models thus cannot handle working memory constraints well (Collins and Frank, 2012). Furthermore, typical RL algorithms assume that agents are explicitly aware of states and actions (cf. Gershman and Niv, 2010).

One recently proposed solution to both how humans infer states and actions from observation and reduce computational demands assumes that humans are constantly updating their beliefs about the latent causes of reinforcement in their environment (Gershman and Niv, 2010). Once structure has been inferred, the agent can then redefine a new set of states to maximize reward. This work fits well with the current study, where humans spontaneously form task-sets comprised of category-response associations, perhaps as a result of inferring structure from the environment (cf. Wilson and Niv, 2012; Rothe et al., 2018). Moreover, this proposal has been expanded to account for differences in memory (cf. Gershman and Daw, 2017; Gershman et al., 2017). Here, associative and structure learning interact such that agents learn conditioned associations between stimuli and responses, but these associations are also informed by the agent’s beliefs about the environment. Whenever an agent learns a new latent cause for a surprising event, a new memory is created, in order to update expectations about the environment; if the surprising event is the result of an old latent cause, memories are updated with additional expectations. In this scenario, while participants were learning about the structure of their environment, new memories are created, but once participants had formed task-sets, old memories were updated even when participants made a surprising error applying their task-sets. This could account for the lack of differences in memory between our learner groups later in the task while explaining early variance in memory due to learning. One open question is whether structure learning has similar effects on memory regardless of what structure (e.g., a task-set, control policy, control state) is being learned.

#### Application Outside the Laboratory

One of the most pervasive demands on modern public health is multi-tasking. Koch et al. (2018) proposed the intriguing notion that task-switching and dual tasking both test common cognitive mechanisms that underlie human multitasking. These mechanisms include processing bottlenecks (structural deficiencies), cognitive flexibility, and cognitive plasticity. Although this experiment was not designed to test multitasking specifically, it may offer an insight into the mechanisms underlying poorer behavioral outcomes while multi-tasking (e.g., Cain et al., 2016; Uncapher et al., 2016). For instance, in the classroom, when students engage in the initial acquisition of new complex rules, teachers cannot and should not expect their students to remember the particulars of the study material rather than just the learned rules. This application of our work outside the laboratory, however, remains an untested speculation to be addressed in the future.

## Conclusion

Adaptive human behavior is characterized by remarkable cognitive flexibility, such that we can adapt to changing demands across environments. One mechanism that facilitates cognitive flexibility is structure learning, whereby we exploit redundancy within our environment to respond more efficiently in line with internal goals. Here, we provide evidence that humans spontaneously form category-response rules, or *task-sets*, but that this tendency toward structure learning also results in a cost to incidental memory. This is one possible mechanism through which multi-tasking leads to poorer behavioral outcomes.

## Data Availability Statement

All datasets generated for this study are included in the article/Supplementary Material.

## Ethics Statement

The studies involving human participants were reviewed and approved by the Institutional Review Board at Duke University. The patients/participants provided their written informed consent to participate in this study.

## Author Contributions

CB and TE developed the study concept and design. CB coded the experiments and collected and analyzed data under the supervision of TE. Both authors contributed to the final version of the manuscript for submission.

## Conflict of Interest

The authors declare that the research was conducted in the absence of any commercial or financial relationships that could be construed as a potential conflict of interest.

## Footnotes

1 http://cabezalab.org/cabezalabobjects/

2 https://osf.io/ixGcd/
