# Supplementary material for: Spontaneous Task Structure Formation Results in a Cost to Incidental Memory of Task Stimuli
Source: Front Psychol. 2019 Dec 17;10:2833. doi: 10.3389/fpsyg.2019.02833 (PMC6929588; doi:10.3389/fpsyg.2019.02833)
Supplement: Supplementary file 1 [file Table_1.docx]

**Supplementary Text**

We here describe an analysis in which we compared the absolute mean switch costs between learner groups with an independent-samples t-test.

**Experiment 1**

**Results and Discussion**

Clustered learners had larger switch costs in RT (*t*(61) = 2.56, *p* = 0.013, Cohen’s *d* = 0.66, CL effect size = 68%) and accuracy (*t*(61) = 1.53, *p* = 0.131, Cohen’s *d* = 0.39, CL effect size = 61%) than non-clustered learners.

**Experiment 2**

**Results and Discussion**

In Experiment 2, clustered and non-clustered learners had mean switch costs that were similar in magnitude (*t* < 1.02). This suggests that when we control for feature priming confounds and when trial-unique images are task-relevant, switch costs vary less as a function of the clustering manipulation.

**Experiment 3**

**Results and Discussion**

Like Experiment 2, the learner groups had post-switch switch costs that were similar in magnitude (RT: *t* *<* 0.15; Accuracy: *t* < 0.73).

**Experiment 4**

**Results and Discussion**

As with Experiment 1, clustered learners had larger RT switch costs than non-clustered learners (*t*(63) = 3.36, *p* = 0.001, Cohen’s *d* = 0.85, CL effect size = 72%; Accuracy: *t*(63) = 1.11, *p* = 0.273, Cohen’s *d* = 0.28, CL effect size = 58%). Because we observed a difference in magnitude between learner groups for Experiments 1 and 4, but not Experiments 2 and 3, this suggests that motor-clustering may increase mean switch costs for superficial and instructed categories, but not for features within highly learned categories (facial features).

*Across Experiments*

To assess the differential effects of instructed vs. implicit task-set learning, we compared switch costs between Experiments 2 and 4 with an ANOVA using experiment (2/4) and learner group (clustered/non-clustered) as between-participants factors. When directly comparing instructed and trial-and-error learning, we found that motor-clustering increased switch costs in RT, but not in accuracy, especially when task-sets were instructed (RT: Experiment x group: *F*(1,125) = 4.64, *p* = 0.033, *η_p_^2^* = 0.04; main effect of group: *F*(1,125) = 10.33, *p* = 0.002, *η_p_^2^* = 0.08; RT and accuracy: main effect of Experiment: *F* < 0.13; Accuracy, group, *F* < 0.01; Experiment x group: *F*(1,125) = 2.25, *p* = 0.136, *η_p_^2^* = 0.02). Altogether, these results suggest that motor-clustering enhances spontaneous switch costs and that this enhancement is strongest for novel categories that are superficially encoded or instructed (i.e., Experiments 1 and 4).

**Table 1. Learning Phase Behavioral Data.**

| **Correct Reaction Time (ms) Switch Costs** | | |
| --- | --- | --- |
|  | Clustered | Non-Clustered |
| *Experiment 1* | 87 [65, 109]** | 54 [40, 67]** |
| *Experiment 2* | 45 [36, 54] | 39 [29, 49] |
| *Experiment 3* | 45 [32, 59] | 47 [35, 58] |
| *Experiment 4* | 58 [44, 72]*** | 28 [18, 39]*** |
|  |  |  |
| **Accuracy (Proportion Correct) Switch Costs** | | |
|  | Clustered | Non-Clustered |
| *Experiment 1* | 0.10 [0.07, 0.14] | 0.08 [0.06, 0.09] |
| *Experiment 2* | 0.07 [0.06, 0.09] | 0.06 [0.04, 0.08] |
| *Experiment 3* | 0.10 [0.07, 0.13] | 0.11 [0.08, 0.14] |
| *Experiment 4* | 0.06 [0.04, 0.08] | 0.07 [0.06, 0.08] |

Confidence intervals are 95%. All switch costs were tested within each group and were p < 0.001. An asterisk here indicates a between-participants comparison, whereby ** p < 0.05; *** p = 0.001.

**Table 2. Memory Phase Behavioral Data**.

|  | | Clustered | Non-Clustered |
| --- | --- | --- | --- |
| **Hit vs. False Alarm**  **(within-participants)** | |  |  |
|  | *Experiment 1* | 0.15 [0.10, 0.20]*** | 0.10 [0.05, 0.15]*** |
|  | *Experiment 2* | 0.18 [0.13, 0.24]*** | 0.16 [0.10, 0.22]*** |
|  | *Experiment 3* | 0.20 [0.14, 0.26]*** | 0.19 [0.13, 0.25]*** |
|  | *Experiment 4* | 0.26 [0.20, 0.32]*** | 0.23 [0.16, 0.29]*** |
| **Hit Rates for category with same button mapping across learner groups**  **(between-participants)** | |  |  |
|  | *Experiment 1* | 0.45 [0.40, 0.51] | 0.48 [0.42, 0.55] |
|  | *Experiment 2* | 0.46 [0.38, 0.54] | 0.46 [0.36, 0.56] |
|  | *Experiment 3* | 0.47 [0.36, 0.57] | 0.53 [0.44, 0.62] |
|  | *Experiment 4* | 0.58 [0.51, 0.66]* | 0.48 [0.39, 0.57]* |

Confidence intervals are 95%. Hit vs. False Alarm data indicate the mean difference between the measures. * 0.10 < p < 0.05; ** p < 0.05; *** p < 0.001
